# Supplementary material for: SWI/SNF catalytic subunits’ switch drives resistance to EZH2 inhibitors in ARID1A-mutated cells
Source: Nat Commun. 2018 Oct 8;9:4116. doi: 10.1038/s41467-018-06656-6 (PMC6175882; doi:10.1038/s41467-018-06656-6)
Supplement: Supplementary file 1 — Supplementary Information [file 41467_2018_6656_MOESM1_ESM.pdf]

SWI/SNF catalytic subunits' switch drives resistance to EZH2 inhibitors in *ARID1A*-mutated cells

Wu S, et al.

Supplementary Figures and Figure Legends

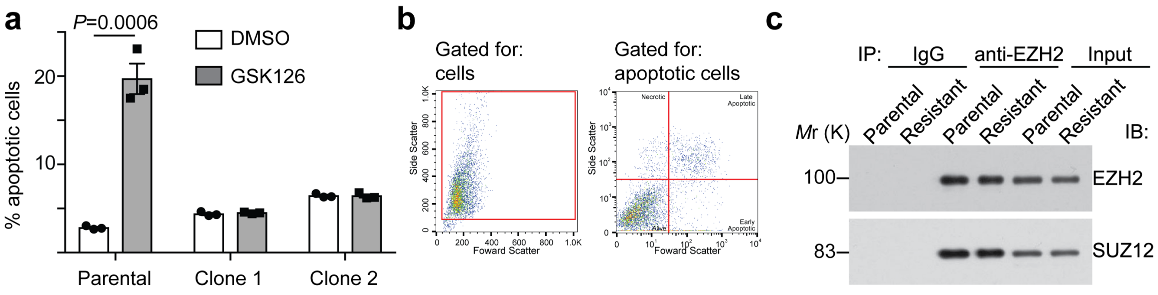

Supplementary Figure 1. EZH2 inhibitor resistant cells are resistant to GSK126 induced apoptosis and there is no change in PRC2 complex stability.

a, Percentage of apoptotic cells in the indicated cells treated with or without 5 μM GSK126 for 72 hours was quantified by Annexin V staining. **b,** The gating strategy used for determining apoptosis based on AnnexinV-FITC and propidium iodide staining. Note that total apoptotic cells are calculated based on both early and late apoptotic fractions. **c**, Co-Immunoprecipitation analysis between EZH2 and SUZ12 for PRC2 complex stability in the indicated cells by using anti-EZH2 antibody or an isotype matched IgG control. Data represent mean ± S.E.M. of three independent experiments. *P*-value was calculated via two-tailed *t*-test.

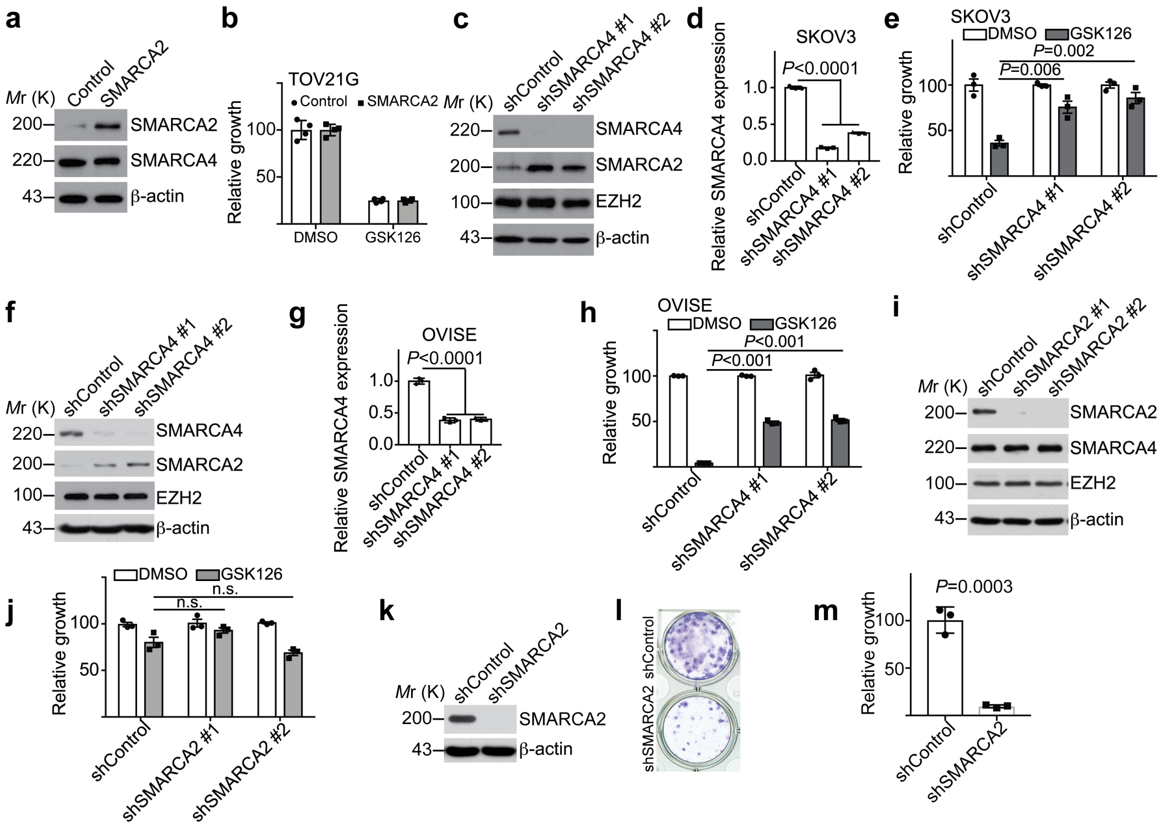

Supplementary Figure 2. SMARCA4 downregulation drives the SMARCA4/SMARCA2 switch that accompanies the *de novo* resistance to EZH2 inhibitors.

a-b, Ectopic SMARCA2 expression in parental *ARID1A*-mutated TOV21G cells does not affect either SMARCA4 levels (**a**) or sensitivity to GSK126 treatment (5 μM) (**b**). **c-e**, SMARCA4 knockdown in *ARID1A*-mutated SKOV3 cells increases SMARCA2 levels (**c-d**) and desensitizes SKOV3 cells to GSK126 treatment (10 μM) (**e**). **f-h**, SMARCA4 knockdown in *ARID1A*-mutated OVISE cells increases SMARCA2 levels (**f-g**) and desensitizes OVISE cells to GSK126 treatment (10 μM) (**h**). **i-j**, SMARCA2 knockdown in EZH2 inhibitor resistant *ARID1A*-mutated TOV21G cells does not affect SMARCA4 levels (**i**) or sensitivity to GSK126 treatment (5 μM) (**j**). **k-m**, Survival of SMARCA2 upregulated EZH2 inhibitor resistant TOV21G cells depends on SMARCA2. SMARCA2 knockdown in EZH2 inhibitor resistant *ARID1A*-mutated TOV21G cells (**i**) suppresses the growth of these cells as determined by colony formation (**l-m**). Data represent mean ± S.E.M. of three independent experiments. *P*-value was calculated via two-tailed *t*-test.

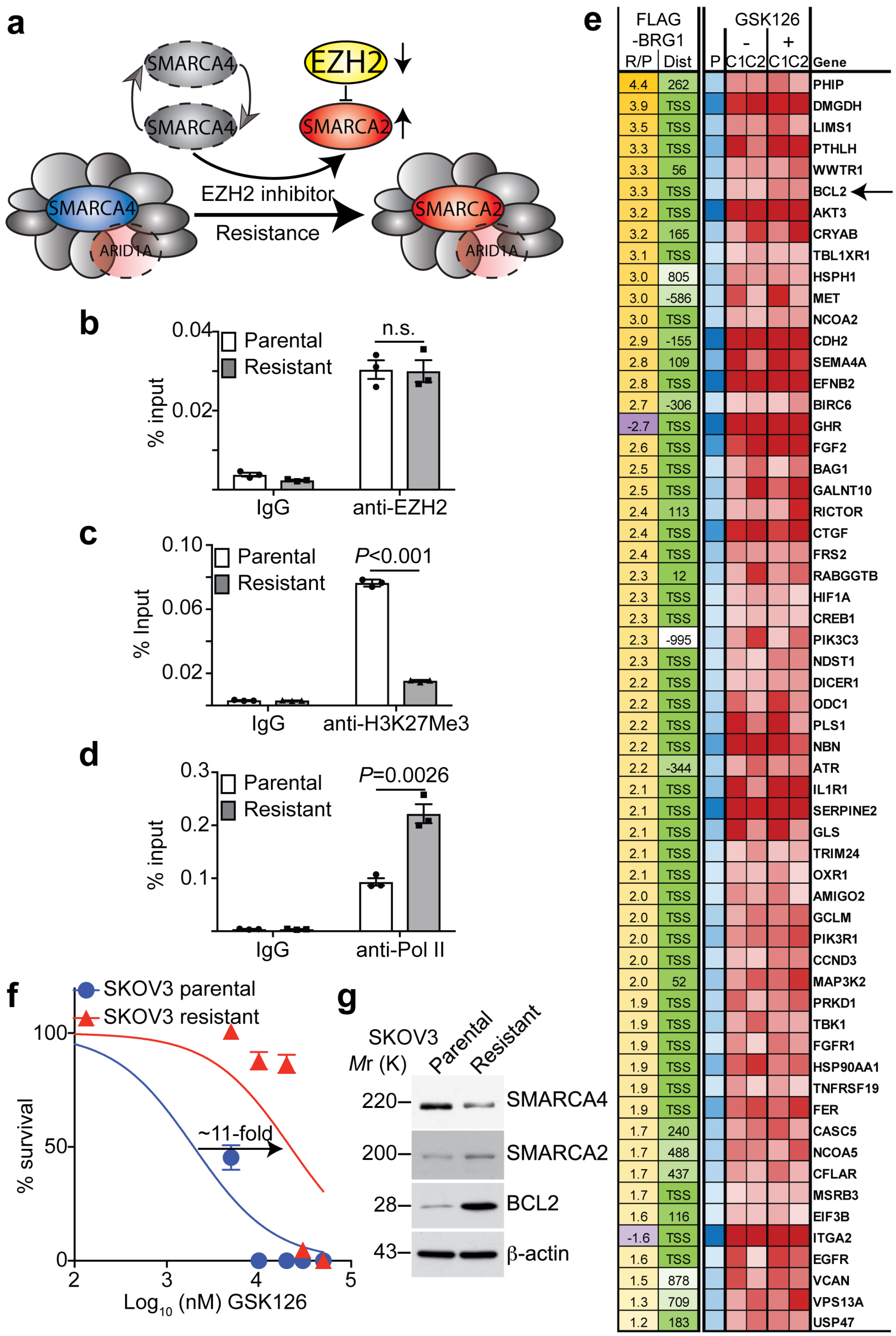

Supplementary Figure 3. SMARCA4 loss promotes resistance by upregulating an anti-apoptosis gene signature.

a, A schematic model: SMARCA4 is downregulated in EZH2 inhibitor resistant cells through a negative feedback loop due to a decrease in the binding of SMARCA4 to its own promoter. SMARCA2 is upregulated in EZH2 inhibitor resistant cells due to loss of EZH2/H3K27Me3 mediated suppression of transcription in its promoter. **b-d,** ChIP-qPCR analysis for the binding of EZH2 (**b**), H3K27Me3 (**c**) and RNA polymerase II (Pol II) (**d**) on the *SMARCA2* gene promoter. **e,** List of direct SMARCA4 target anti-apoptotic genes that are upregulated in EZH2 inhibitor resistant cells passaged in medium with or without GSK126. P: parental cells; C1 and C2, two independent EZH2 inhibitor resistant *ARID1A*-mutated TOV21G clones. Data represent mean ± S.E.M. of three independent experiments (**b-d**). *P*-value was calculated via two-tailed *t*-test. **f-g,** Parental and GSK126 resistant *ARID1A*-mutated SKOV3 cells were treated to generate dose response curves to GSK126 (**f**). Arrow points to an ~11-fold increase in GSK126 IC_{50} in the resistant cells. Expression of SMARCA4, SMARCA2, BCL2 and a load control β-actin in the indicated cells determined by immunoblot (**g)**.

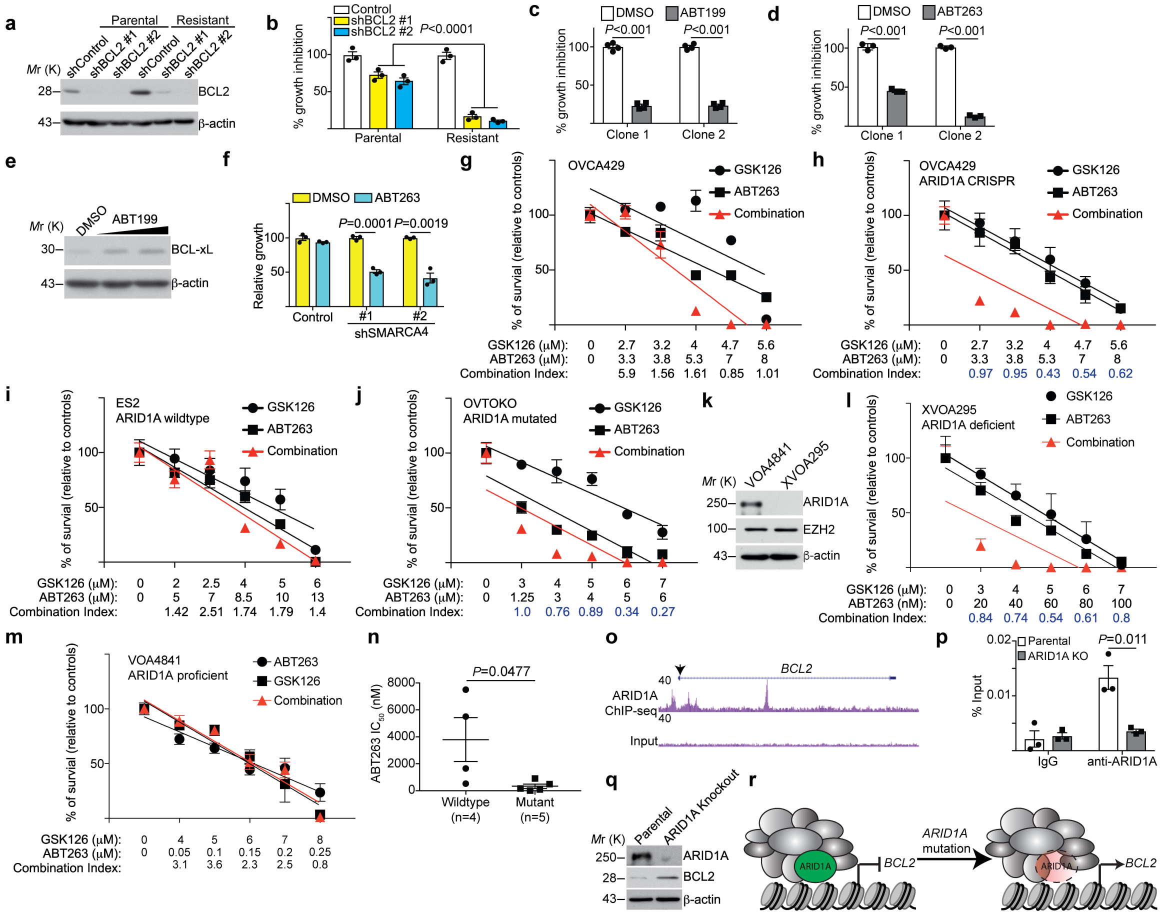

Supplementary Figure 4. EZH2 inhibitor resistant cells are sensitive to inhibition of anti-apoptotic BCL2.

a-b, EZH2 inhibitor resistant cells are hypersensitive to BCL2 knockdown. BCL2 knocking down in parental and resistant cells (**a**) were subjected to growth analysis by cell counting (**b**). **c-d,** Effect of BCL2 inhibitors 5 μM ABT199 (**c**) or 0.5 μM ABT263 (**d**) on the growth of EZH2 inhibitor resistant cells determined by colony formation. **e,** Expression of BCL-xL in EZH2 inhibitor resistant cells post ABT199 treatment (5 μM and 10 μM ABT199) for 48 hours. **f**, SMARCA4 knockdown sensitizes parental TOV21G cells to ABT263 (0.25 μM ABT263). Note that the low concentration of ABT263 used here did not significantly affect the growth of parental TOV21G cells. **g-j,** Synergy analysis between GSK126 and ABT263 in *ARID1A* wildtype OVCAR429 (**g**), ARID1A knockout OVCAR429 (**h**), *ARID1A* wildtype ES2 (**i**) and *ARID1A*-mutated OVTOKO (**j**) cells. **k,** Expression of ARID1A, EZH2 and β-actin in the indicated primary OCCC cultures determined by immunoblot. **l-m,** Synergy analysis between GSK126 and ABT263 in ARID1A deficient (**l**) and proficient (**m**) primary OCCC cultures. **n,** IC_{50} of ABT263 in *ARID1A* wildtype and mutated cells. **o,** ARID1A ChIP-seq and input track on the *BCL2* gene promoter in *ARID1A*-wildtype RMG1 cells [1] (GEO access number:

GSM2803052). **p-q**, Validation of binding of ARID1A to the *BCL2* gene promoter in ARID1A wildtype RMG1 control and ARID1A knockout RMG1 cells by ChIP-qPCR analysis (**p**) and upregulation of BCL2 by immunoblot analysis (**q**). **r,** Schematic model showing ARID1A inactivation upregulates BCL2. Data represent mean ± S.E.M. of three (**b, d, f, g-l**) or four (**c**) independent experiments. *P*-value was calculated via two-tailed *t*-test.

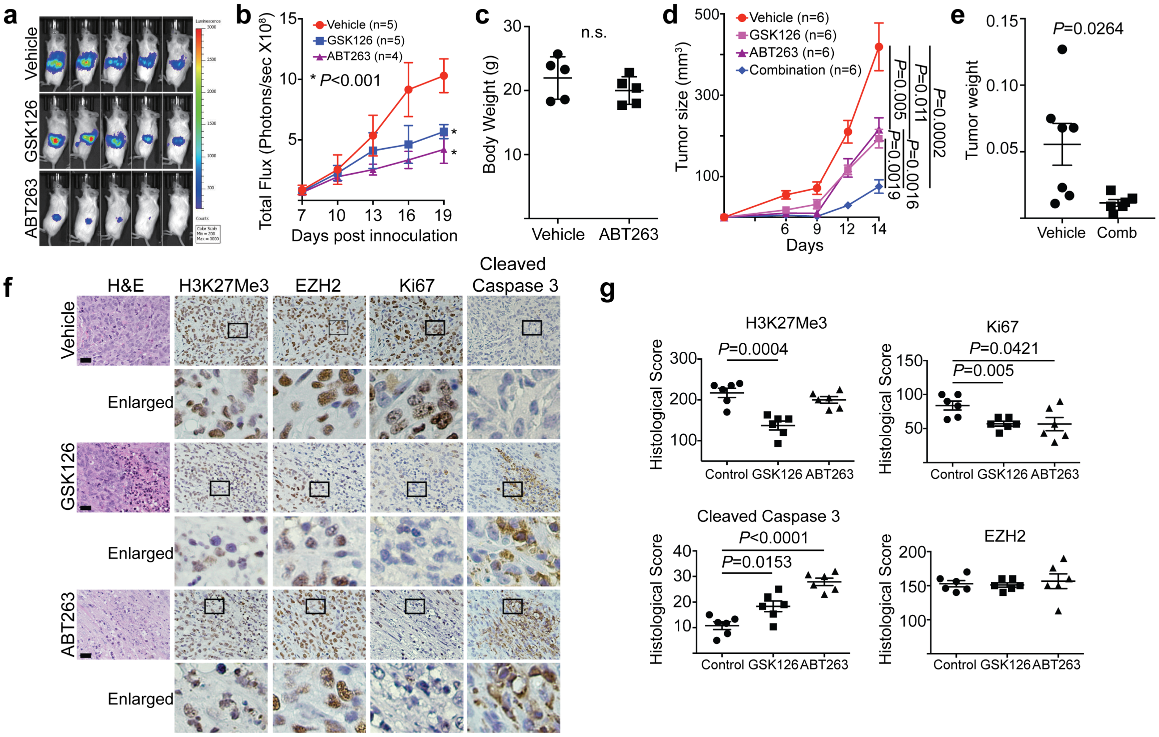

Supplementary Figure 5. ABT263 overcomes resistance to EZH2 inhibitor in *ARID1A*-mutated tumours *in vivo*.

a, Luciferase-expressing *ARID1A*-mutated EZH2 inhibitor resistant TOV21G cells were orthotopically transplanted into the ovarian bursa sac of 6-8 weeks old NSG female mice. Tumours were allowed to establish for three weeks before randomized into three groups (n=5 mice/group). Mice were treated with vehicle control, GSK126 (50 mg/kg, daily) or ABT263 (50 mg/kg, daily) for two weeks. Representative luminescent images of control and treated mice at the end of treatment. **b**. Luciferase-expressing *ARID1A*-mutated EZH2 inhibitor sensitive parental TOV21G cells were orthotopically transplanted into the ovarian bursa sac of SCID/nude female mice. Tumours were allowed to establish for 7 days before randomized into three groups. Mice were treated with vehicle control (n=5 mice), GSK126 (50 mg/kg, n=5 mice) or ABT263 (50 mg/kg, n=4 mice) daily for 12 days. Total flux (photons/sec) is graphed at the indicated time points. **c,** Same as a, but the body weight of mice from the indicated groups were examined at the end of treatment (n=5 mice/group). **d**, ARID1A-deficient XVOA295 primary OCCC cultures were subcutaneously injected into 6-8 weeks old NSG female mice. Mice were randomized into 4 groups (n=6 per group) and treated with vehicle control, GSK126 (50 mg/kg), ABT263 (50 mg/kg) or a combination. Tumour growth was measured twice a week. **e**, 6-10 weeks old *Pik3ca*^{H1047R}/ *Arid1a*$^{flox/flox}$ female mice were intrabursally injected with adenovirus-Cre to induce clear cell ovarian carcinomas. Mice were randomized and treated with vehicle control (n=7 mice) or a combination of GSK126 (50 mg/kg) and ABT263 (50mg/kg, n=6 mice) daily for 21 days. The weight of dissected

tumours from the indicated groups at the end of experiment was measured as a surrogate for tumour burden. **f-g,** Immunohistochemistry staining of parental TOV21G orthotopic tumours for EZH2, H3K27me3, Ki67 and cleaved caspase-3 using consecutive sections of the dissected tumours from the indicated treatment groups (**f**). Bar = 20 μm. Histological score (H score) calculated for the indicated staining (**g**). *P*-value was calculated via two-tailed *t*-test.

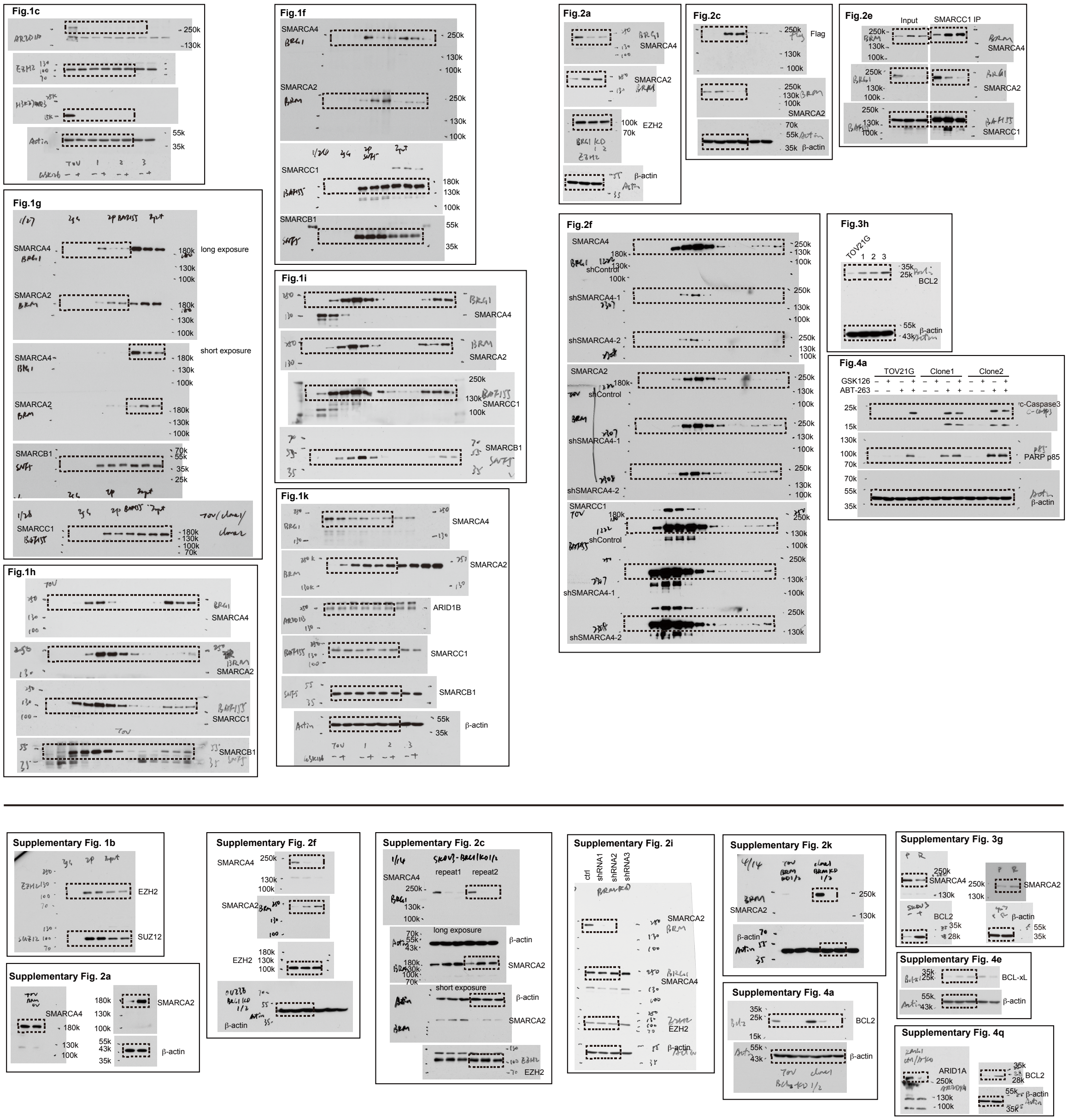

Supplementary Figure 6. Unprocessed images of blots. Unprocessed images of scanned immunoblots shown in Figures and Supplementary Figures are provided.

Supplementary Table and Table Legends

IC50	EZH2 inhibitors		Chemotherapeutics	
	EPZ6438	CPI169	Paclitaxel	Cisplatin
Parental	370 nM	400 nM	1.9 nM	121 nM
Resistant	2900 nM	6900 nM	2.0 nM	138 nM

Supplementary Table 1. IC_{50} of the listed EZH2 inhibitors and chemotherapeutic agents in parental and EZH2 inhibitor resistant *ARID1A*-mutated TOV21G cells.

Supplementary Reference

1. Trizzino M*, et al.* The Tumor Suppressor ARID1A Controls Global Transcription via Pausing of RNA Polymerase II. *Cell reports* **23**, 3933-3945 (2018).
